# Obesity in total hip arthroplasty—does it really matter?

**DOI:** 10.3109/17453674.2011.652893

**Published:** 2012-02-08

**Authors:** Anne Lübbeke

**Affiliations:** Division of Orthopaedic Surgery and Traumatology, CH-1211 Geneva, SwitzerlandAnne.LubbekeWolff@hcuge.ch

*Sir*—I read with great interest the meta-analysis “Obesity in total hip arthroplasty—does it really matter?” by Haverkamp et al. published in the August 2011 issue. However, it seems to me that there is an error in their analysis regarding the outcome “aseptic loosening”.

**Figure 4.**
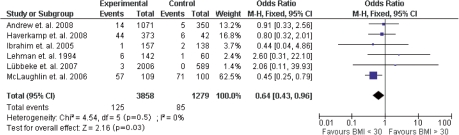
Forest plot, aseptic loosening.

Figure 4. Forest plot aseptic loosening (below) contains wrong numbers producing a wrong overall result and wrong study conclusions with respect to revision for aseptic loosening (abstract and result section).

The numbers in the study by McLaughlin et al. (2006) should read: 71 – 109 – 56 – 100. (I assume that the column “Experimental” should read “BMI < 30” and the column “Control” “BMI > 30” as in the other figures).

The resulting OR for this study should then be 1.16 (95% CI 0.9; 1.5) instead of 0.45. And as a consequence, the total events and total OR are incorrect as well.

Furthermore, there are three other issues I would like to address:

1. The study by Andrew et al. (2008) included in the analysis for Figure 4 does not specify the reason for revision, and the numbers of revision indicated here (14 in non-obese and 5 in obese) can theoretically also contain cases with revision for septic loosening, dislocation or other.
2. A large study (n = 2026 primary THAs) conducted by Jackson et al. (2009) with the title “The effect of obesity on the midterm survival and clinical outcome of cementless total hip replacement” has not been included in the meta-analysis.
3. The authors write in their discussion (page 441, 2. Paragraph): “Previous studies have suggested that dislocation occurs more often in obese people (Paterno et al. 1997, Sadr et al. 2008).” However, the study by Paterno et al. does not report on a higher risk in obese. He found a dislocation risk of 3% in obese vs. 5% in non-obese patients. To the best of my knowledge the studies by Lübbeke et al. (2007) and Sadr et al. (2008) were the first to demonstrate a significantly higher rate of dislocation in obese patients.
