# Supplementary material for: Obesity in total hip arthroplasty—does it really matter?
Source: Acta Orthop. 2012 Feb 8;83(1):99–100. doi: 10.3109/17453674.2011.652893 (PMC3278665; doi:10.3109/17453674.2011.652893)
Supplement: Related texts

# Letter: Obesity in total hip arthroplasty—does it really matter?

**Published:** 2012-02-08

**Authors:** D. Haverkamp

**Affiliations:** Department of Orthopaedic Surgery, NL-1100 DD Amsterdam, The NetherlandsDaniel@drhaverkamp.com

*Sir*—We would like to thank Dr Lübbeke for her critical analysis of our manuscript. The main concern is the interpretation of the aseptic loosening in our meta-analysis and our conclusion regarding the occurrence of aseptic loosening in obese compared to non obese.

The study of McLaughin et al. (2006) shows no difference between obese and non-obese with regard to the long term outcome, however in both groups more than 55% were revised for loosening at the time of analysis. We agree that we made a mistake in the data extraction, and that the Mclaughin study was not correctly cited. The small difference that seems to be in the disadvantage of the obese disappears, the correct odds ratio is 1.2 (95% CI 0.78–1.8). Since the study of Mclaughin has 50% of the weight in our comparison, based on the large number of revisions, we also made an analysis without this study showing a similar outcome (OR 0.97 (95%CI: 0.53–1.77). Therefore it cannot be said that obese have a higher risk of revision for aseptic loosening as stated earlier.

In the study of Andrew et al. (2008) it is correct that the reason for revision is not specified as being aseptic, however all the complications like deep infection and dislocation are mentioned separately and for none it is mentioned that this resulted in a revision surgery. Based on a critical analysis of this manuscript we assumed that the revisions mentioned were performed for aseptic loosening. Leaving this study out of the pooling did not change the abovementioned outcome.

The study of Jackson et al. (2009) concerns the survival of THA in the obese compared to the none obese and this study appears valid to be included in our analysis. Data extraction results in 8 revisions in 1,652 (0.5%) non-obese and 3 revisions in 414 (0.7%) obese for aseptic loosening. Including this in the meta-analysis changes OR to 1.1 (0.8–1.7).

The main concern of Dr Lübbeke is correct, we cannot prove a difference in aseptic loosening in obese versus non-obese. Based on the current evidence it cannot be excluded either. The main concern is the large heterogeneity between the studies; comparing revision rates of 65% (McLaughin) and 0.5% (Jackson) is difficult.

Regarding the last remark, we obviously cited the wrong article in the discussion. While Lübbeke et al. and Sadr et al. are mentioned in the text and should be cited, we cited Paterno instead of Lübbeke. Of course Dr Lübbeke was the first to show the difference in dislocation rate between obese and non-obese.

The main outcome of our study was a higher infection rate in obese compared to non-obese and a higher dislocation rate in obese, no doubt about this. Regarding the occurrence of aseptic loosening we did not find a difference between obese and non-obese.

## References

[CIT0001] Andrew JG, Palan J, Kurup HV, Gibson P, Murray DW, Beard DJ (2008). Obesity in total hip replacement. J Bone Joint Surg (Br).

[CIT0002] Jackson MP, Sexton SA, Yeung E, Walter WL, Walter WK, Zicat BA (2009). The effect of obesity on the mid-term survival and clinical outcome of cementless total hip replacement. J Bone Joint Surg (Br).

[CIT0003] Lubbeke A, Stern R, Garavaglia G, Zurcher L, Hoffmeyer P (2007). Differences in outcomes of obese women and men undergoing primary total hip arthroplasty. Arthritis Rheum.

[CIT0004] McLaughlin JR, Lee KR (2006). The outcome of total hip replacement in obese and non-obese patients at 10- to 18-years. J Bone Joint Surg (Br).

[CIT0005] Paterno SA, Lachiewicz PF, Kelley SS (1997). The influence of patient-related factors and the position of the acetabular component on the rate of dislocation after total hip replacement. J Bone Joint Surg (Am).

[CIT0006] Sadr AO, Adami J, Lindstrom D, Eriksson KO, Wladis A, Bellocco R (2008). High body mass index is associated with increased risk of implant dislocation following primary total hip replacement: 2,106 patients followed for up to 8 years. Acta Orthop.
